# Family Trouble

**Published:** 1872-10

**Authors:** 


### HALL'S

### JOURNAL OF HEALTH.

VOL. XIX.—OCTOBER, 1872.—NO. X.

### FAMILY TROUBLE

Is the fruitful cause of disease and premature death; one fourth of the suicides in France are the result of this avoidable misfortune, bringing blasting and blight and mildew to many a flourishing household; and there is reason to believe that in prosperous and happy New England, where the disgrace of divorce is becoming so common that it is ceasing to be disgraceful, the first foundation stones are laid by the wives of the land. At first sight, such a statement would seem to be as unjust as it is absurd and monstrous. But when it comes to the official facts of the case, that forty, and fifty, and even more divorces are asked for at a single court, in educated, moral, and religious New England, it is worth while to seek for a cause adequate to the state of the case. It would not be a fair solution of the difficult problem to determine whether husbands or wives were the most numerous applicants; nor to ascertain the most frequent grounds on which divorce was sought and obtained; it can only be truthfully decided by having the opportunity to cross-question the applicants and trace backwards to the very fountain-head, the spring, the first cause of disquietude. In our book just published, entitled

"HEALTH AT HOME,"

the subject is more fully discussed, and the different grounds named. The most frequent by all odds cannot well be stated in a monthly for promiscuous reading; but one of the grounds becomes so apparent, in going the rounds of the leading summer watering-places, that it may be of some general importance to throw out a few hints, to be pondered over by husbands and wives, between this and the summer of the next year. In visiting the public places of resort of the first and second class in New England, two features are pressed upon the attention; first, the uniformly crowded state of these places; second, the preponderance in numbers of women. The men are at home, attending to business. At Wolfsboro', on the beautiful Winnipiseogee, of sixteen persons in the dance, there were only two men. At this present writing, at one of the most magnificent and varied sea views on all the Atlantic coast, the gentlemen are in the proportion of one to ten. From facts like these, and others naturally coming to the notice of physicians in large cities, in the way of confidential communications, it may be very safely stated that. in very many cases of divorce, the first stone is cast by the wife, in two ways: one is the easy separation from husbands for a night, or week, or month, or year; the other, giving six divorces out of ten, but being private, is not stated here. It is "thought nothing of" now, for a wife, especially a wife and grown daughter, to go abroad alone for a season, or a year or more; or to be taken there and left by the husband, he returning home to business, they to make their way back in a year or two; this is simply monstrous. At home, a wife or mother has her mind and attention fully taken up with household cares and responsibilities; commencing in the early morning, having no surcease until the "hour for retiring." With the husband it is very different. It is true that he is quite as absorbingly engaged in business, but only until the evening; then, to come home to a solitary fireside, wearied in body, discouraged, or anxious, or annoyed, or dispirited in mind, the very quietude is an aggravation; for there is nothing to lift the thoughts out of the rut in which they have been running all day, and thus the brain reels in

"ONE ETERNAL ROUND,"

the irritation and the aggravation constantly increasing at each revolve, until, in multitudes of cases, it amounts to a frenzy, and the restless spirit hurries the man out of the door, he scarcely knowing or caring whither. He must go somewhere, and instinctively steps in where there is welcome and light and sounds of cheer; anything, anywhere, for a change, to get out of the influence and power and control of the demon brooding within. It is not without reason that the bar-room and the drinking saloon have always an open door, wide swinging; not without reason that the entrance and the surroundings are made as inviting as paint and gaslight and other adornments can make them. The dirtiest groggery has sometimes the green cedar entwined over the door, or a tiny tree or branching bough, freshly broken off, at either side; to these, music, soft and sweet, is not unfrequently added; and when once the head has bowed to the lintel, and the feet have crossed the threshold, desperation and death and hell run riot and rampant. Wives of the land, what are you thinking about? Are you demented? or have you ceased to think at all? That man tempts destruction to himself, who marries a woman when he knows his business calls him often from home; or who accepts of a position after marriage, which compels frequent and even short absences from his own table and fireside and bedchamber. Many a man is not an actual thief, for want of an opportunity of thieving with a certainty of not being found out here below. So, many are moral and virtuous and temperate, only because they have not been confronted with what is wrong and vicious and unclean. The boy who usually makes his way into the street after nightfall, is pretty certain to "fetch up" at the penitentiary or at the lower end of a halter. This is because home, the fireside, was not made more attractive by a mother's loving and saving influences. It is not a whit less true that if a man finds less cheer in his own home than at the club, the bar, and, lower down, at the corner grocery, his love and respect for the woman of his first love will be absorbed in the degrading attractions and allurements of the bottle, the card-table, and lower down still.

The wise wife should always bear in mind that although the husband is the oak of the family, he is largely sustained by the little tendrils of conjugal sympathy, encouragement, and praise; by these he is made brave and strong and persistent in the prosecution of his business, whether it be the tinkering of a tin pan, the mending of a shoe, or the building of a steamship. It is impossible for any woman to overrate the importance of these suggestions. A new and big word may make it more impressive. It is an idea which is the very

ANTIPODIZATION

of all physical law. The jasmine and the morning-glory wind around the sturdy oak of a hundred winters; they adorn its roughened bark, and give out sweet odors all around, receiving pay in the ability which is afforded them of withstanding the fury of the wildest tempest, and of the tornado; but in the married relation, in the connection already referred to, it is the tendril which supports the oak. A wife's condolence and commiseration in the mishaps of business; her coöperation, mental, moral, and physical; her cordial unity and sympathy in those projects and enterprises which are constantly presenting themselves in the work of life, do more than anything else to impart force, to infuse energy, and to insure success in the business engagements of the husband; and more than at any other time does he need these, does he look for them, when he comes home at the close of a day's battle with the selfishness and trickeries and hardness of the world around him, wearied and worn, and but too often almost exhausted in the conflict. A tender word, some little trifling, considerate, loving act, under such circumstances, has many a time waked up a man to life again, to renewed resolves, more efficiently than half a glass of brandy; he was faint, almost to falling; something he must have, at least, so he thinks; in millions of cases, alas! when he failed to get the loving word, he took instead the fiery glass, and on the instant, he was a lost man.

A few men, one in tens of thousands, can stand, and do stand, without any helps like these; but even they could have stood the better with them.

Others there are who get along awhile without them, but with the increasing heat and burden of the day, they faint and fail and fall by the way.

Other men, after a season, are made desperate by an absence of coöperation and sympathy and encouragement, from their wives; the disposition is embittered, the whole nature is soured; the affections are exterminated; the fireside invites no more; outside excitements are sought; other associations are formed, and there is no home in that house evermore.

In countless cases, these things have taken place from the mere absence of wifely qualifications. But when, instead of this, there is an aggressive conduct; when dissatisfaction and complaining and fault-finding are the rule, until a man has endured so long, that he expects nothing else on his arrival but to be met with frowns and impatience, one of two results is inevitable always; domestic joy flees the household forever, or there opens the drunkard's grave, the door of the criminal, or the gate of the broad road to a blasted, blighted life.

Let wise wives meditate on these things, and let conscientious mothers seek to impress them early and persistently on their unmarried daughters; it will be an unfailing spring of domestic peace and beatitude; a legacy worth more than millions of money. At the same time, let every husband of intelligence and culture and manliness remember; let the farmer and mechanic and daily laborer remember; that to their patient, toiling, and too often overtaxed wives, are due a still more liberal share of *their* sympathy, consideration, loving attentions, and abnegations, always due to those whom, at the marriage altar, they have sworn to cherish "so long as they both shall live;" in all things, and at all times and places, showing to servants and children and friends and guests and strangers, that the wife is the queen of the household, and merits all the affection and respect which any of them can show to her.

